# Supplementary material for: GDNF Increases Inhibitory Synaptic Drive on Principal Neurons in the Hippocampus via Activation of the Ret Pathway
Source: Int J Mol Sci. 2022 Oct 29;23(21):13190. doi: 10.3390/ijms232113190 (PMC9653719; doi:10.3390/ijms232113190)
Supplement: Supplementary file 1 [file ijms-23-13190-s001.zip › ijms-1968572-supplementary.pdf]

Supplemental Information

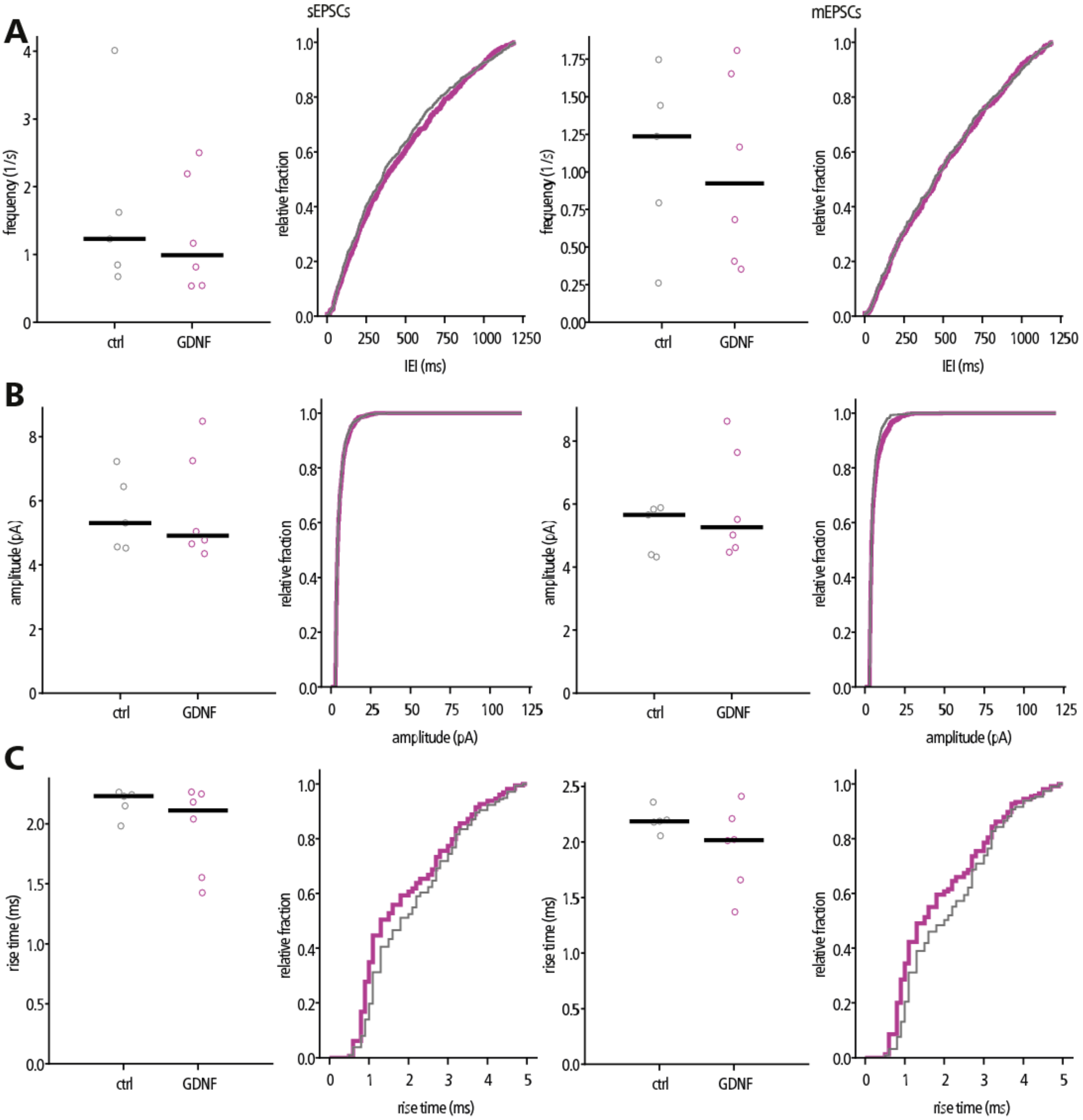

Figure S1. Quantification of GDNF effect on excitatory post-synaptic currents. (A) Inter-event interval (K-S sEPSCs $p<0.01$, $D=0.086$, mEPSCs $p<0.01$, $D=0.099$), (B) amplitude (K-S sEPSCs $p>0.01$, $D=0.033$, mEPSCs $p>0.01$, $D=0.070$) and (C) rise-time (K-S sEPSCs $p<0.01$, $D=0.151$, mEPSCs $p<0.01$, $D=0.154$) cumulative distribution plots of spontaneous (left) and miniature EPSCs (right) from control and GDNF-incubated slices (n = 201 sEPSC events, n = 140 mEPSC events per cell). The line markers in the scatter plots depict the median of averages per cell. Mann-Whitney tests for the averages not significant.

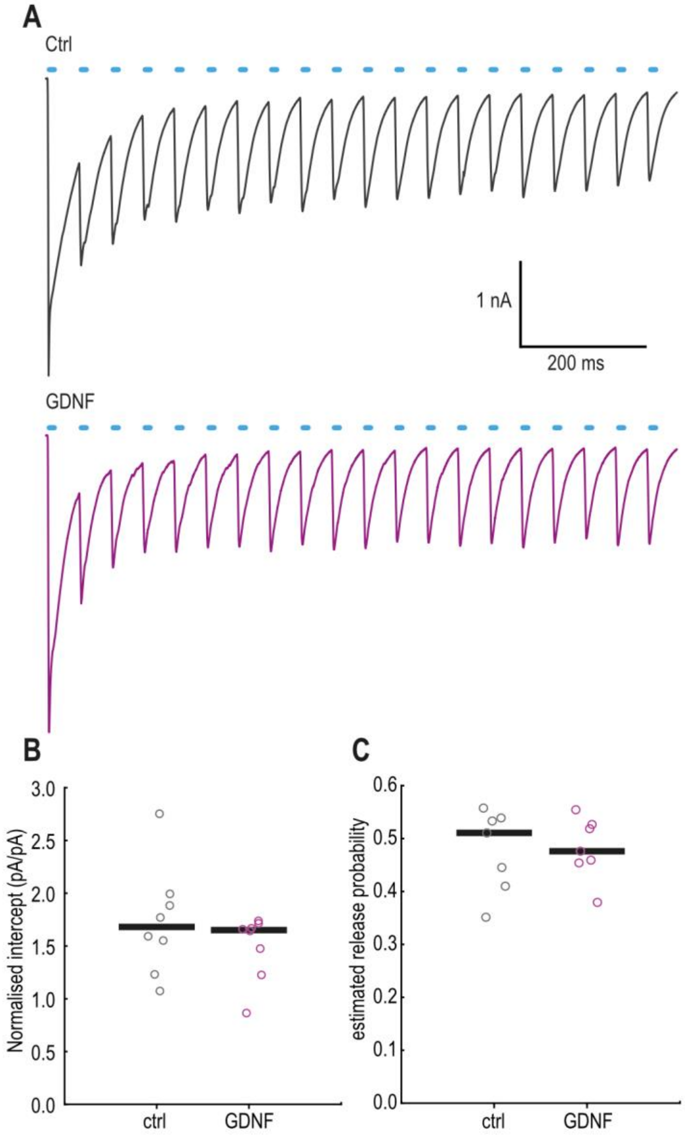

Figure S2. GABA release properties estimation. (A) example averaged traces of light-pulse train responses from a CA1 pyramidal neuron in a control aCSF-incubated slice (top) and a CA1 pyramidal neuron in aCSF with GDNF (bottom). Blue lines indicate the timing of light stimulation (3 ms blue light pulses). (B) Normalised SMN-fit intercept values for control slice (n=8) and GDNF-incubated slice (n=8) cells (M-W U test p = 0.3823, ns). (C) Release probability estimates for control and GDNF cells for control slice (n=7) and GDNF-incubated slice (n=7) cells (M-W U test p = 1.000, ns)

Table S1. Comparison of EPSC averages ± standard error of mean by cell following control and GDNF incubation for 1h.

	Frequency (Hz)		Amplitude (pA)		Rise time (ms)	
	sEPSCs	mEPSCs	sEPSCs	mEPSCs	sEPSCs	mEPSCs
Ctrl	1.7 ± 0.5, n=5	1.1 ± 0.2, n=5	5.6 ± 0.4, n=5	5.2 ± 0.3, n=5	2.17 ± 0.04, n = 5	2.19 ± 0.04, n=5
GDNF 2nM	1.3 ± 0.3, n=6	1.0 ± 0.2, n=6	5.7 ± 0.6, n=6	6.0 ± 0.6, n=6	1.95 ± 0.14, n = 6	1.95 ± 0.14, n=6

Mann-Whitney p	0.261	0.464	0.464	0.324	0.324	0.158

Table S2. Series and input resistance, and resting membrane potential for the cells used for the IPSC recordings.

	R_s (MOhm)		R_i (MOhm)		V_{mr} (mV)	
	sIPSCs	mIPSCs	sIPSCs	mIPSCs	sIPSCs	mIPSCs
Ctrl	10.6 ± 1.63, n=7	15.5 ± 2.5, n=7	173.7 ± 18.7, n=7	274.8 ± 55.5, n=7	-66.1 ± 0.2, n=7	-65.6 ± 0.1, n=7
GDNF 2nM	12.1 ± 2.1, n=6	17.8 ± 2.7, n=8	189.4 ± 16.5, n=6	193.9 ± 16.6, n=8	-66.1 ± 0.3, n=6	-65.6 ± 0.1, n=8
Mann-Whitney p	0.415	0.226	0.216	0.343	0.500	0.157

Table S3. Series and input resistance, and resting membrane potential for the cells used for the EPSC recordings.

	R_s (MOhm)		R_i (MOhm)		V_{mr} (mV)	
	sEPSCs	mEPSCs	sEPSCs	mEPSCs	sEPSCs	mEPSCs
Ctrl	18.2 ± 2.3, n=4	17.2 ± 2.2, n=4	340.4 ± 63.8, n=4	366.0 ± 47.8, n=4	-66.2 ± 0.2, n=4	-65.6 ± 0.1, n=4
GDNF 2nM	15.2 ± 3.4, n=5	14.6 ± 1.5, n=6	199.8 ± 34.6, n=5	230.3 ± 46.1, n=6	-65.6 ± 0.4, n=5	-65.8 ± 0.1, n=6
Mann-Whitney p	0.357	0.169	0.070	0.083	0.054	0.139

Table S4. Series and input resistance, and resting membrane potential for the cells used for the XIB4035 recordings.

	R_s (MOhm)		R_i (MOhm)		V_{mr} (mV)	
	sIPSCs	mIPSCs	sIPSCs	mIPSCs	sIPSCs	mIPSCs
GDNF	16.2 ± 2.7, n=9	21.6 ± 3.1, n=8	156.9 ± 25. 7, n=9	177.5 ± 25.2, n=8	-66.2 ± 0.3, n=9	-65.8 ± 0.1, n=8

GDNF + XIB4035	16.8 ± 2.8, n=11	16.2 ± 2. 6, n=7	178.8 ± 20.8, n=11	266.1 ± 46.2, n=7	-66.0 ± 0.1, n=11	-65.7 ± 0.1, n=7
Mann-Whitney p	0.440	0.112	0.311	0.209	0.380	0.500
